# A first-in-human study of the anti-α5β1 integrin monoclonal antibody PF-04605412 administered intravenously to patients with advanced solid tumors

**DOI:** 10.1007/s00280-014-2576-8

**Published:** 2014-09-12

**Authors:** J. Mateo, J. Berlin, J. S. de Bono, R. B. Cohen, V. Keedy, G. Mugundu, Lianglin Zhang, A. Abbattista, C. Davis, C. Gallo Stampino, H. Borghaei

**Affiliations:** 1 The Royal Marsden NHS Foundation Trust and The Institute of Cancer Research, Sutton, UK; 2 Vanderbilt-Ingram Cancer Center, Nashville, TN USA; 3 Developmental Therapeutics Program and Department of Medical Oncology, Fox Chase Cancer Center, 333 Cottman Ave, Philadelphia, PA 19111 USA; 4 Pfizer Oncology, La Jolla, CA USA; 5 Pfizer Oncology, Groton, CT USA

**Keywords:** Integrin, Antibody, Angiogenesis, ADCC, Phase I trial, First-in-human

## Abstract

**Purpose:**

A first-in-human clinical trial of a fully human, Fc-engineered IgG1 monoclonal antibody targeting integrin α5β1 was conducted to evaluate tolerability, maximum tolerated dose, pharmacokinetics, pharmacodynamics and preliminary anti-tumor activity.

**Methods:**

Escalating doses of PF-04605412 were given IV on day 1, 28 and every 2 weeks thereafter to patients with advanced solid tumors until disease progression or unacceptable toxicity. Sequential dose cohorts were evaluated based on a modified 3 + 3 dose-escalation design. The starting dose was 7.5 mg based on preclinical data.

**Results:**

Thirty-three patients were enrolled to six dose levels (7.5, 11.25, 16.9, 34, 68 and 136 mg). Twenty-three patients were evaluable for the primary endpoint (determination of the maximum tolerated dose). Five patients required permanent drug discontinuation due to acute infusion-related reactions, which occurred as grade 3 events in two patients. PK analysis indicated that the targeted drug exposure based on preclinical models was not achieved by the tolerated doses and PK modeling suggesting that doses at least fivefold higher would be necessary. No anti-tumor activity was observed.

**Conclusion:**

Based on the safety data, the risks associated with the likelihood of significant cytokine-mediated infusion reactions at higher doses, the projected high dose necessary to affect on the biological target and the lack of anti-tumor activity at the doses explored, the trial was prematurely terminated without determining a formal maximum tolerated dose. Further clinical development of PF-04605412 has been discontinued.

**Electronic supplementary material:**

The online version of this article (doi:10.1007/s00280-014-2576-8) contains supplementary material, which is available to authorized users.

## Introduction

Integrins are a family of transmembrane glycoprotein receptors that provide docking sites for endothelial and inflammatory cells and thereby regulate interactions between the cell and the extracellular matrix. Integrins also play a key role in signal transduction [1, 2]. Each integrin is composed of an α and a β transmembrane subunit, which combine to form more than 20 distinct integrins that are expressed in malignant and normal cells. The specific pairing of α/β subunits defines the function of the protein [3, 4].

Given the role of integrins in cancer cell proliferation, invasion, metastasis and angiogenesis, they are considered a potential target in anticancer drug development. One obvious challenge for targeting integrins in oncology is their widespread expression in non-cancerous cells [5]. Integrin α5β1 plays key roles in cell adhesion, migration, proliferation, differentiation, and survival in both normal and tumor cells [6–8]. In human cancers, the expression of integrin α5β1 and fibronectin is significantly and coordinately enhanced on tumor blood vessels and in tissues stimulated by growth factors and cytokines. Integrin α5β1 is also frequently observed in many types of cancer and tumor-associated macrophages and fibroblasts. Preclinical data with agents that disrupt the functions of integrin α5β1 support its targeting as an anti-angiogenic strategy for the treatment of human malignancies [9, 10].

Clinical data have been previously reported for volociximab (M200, PDL Biopharma and Biogen Inc), a chimeric (82 % human, 18 % murine) IgG4 monoclonal antibody (mAb) against α5β1 integrin. Overall good tolerance, with minor constitutional adverse events, was described in a phase I study testing doses up to 15 mg/kg QWK [11]. Although single-agent anti-tumor activity was disappointing [12], the good safety profile led to a trial combining volociximab with carboplatin/paclitaxel in non-small cell lung cancer [13].

PF-04605412 is a fully human, Fc-engineered anti-α5β1 IgG1 mAb with anti-angiogenic properties. PF-04605412 potently inhibited integrin α5β1-mediated cell spreading, adhesion and tube vessel formation, and induced significantly greater antibody dependent cellular cytotoxicity (ADCC) against endothelial cells or α5β1-bearing tumor cells when compared with wild-type IgG1 anti-α5β1 mAb. PF-04605412 exhibited robust single-agent anti-angiogenesis and anti-tumor properties, with regression in tumor size and prolongation of survival in xenograft models, which was associated with macrophage tumor infiltration, increased caspase-3 and decreased Ki67 signal in the tumor. The mechanism of action of PF-04605412 was studied in transgenic knock-in mouse models in which the coding sequence of integrin α5 was replaced by the human counterpart, with demonstration of dose-dependent tumor growth inhibition [14]. Proof of principle for ADCC has been demonstrated for several monoclonal antibodies currently in clinical use including rituximab, alemtuzumab, trastuzumab and cetuximab [15–22]. In order to optimize the interaction between antibody Fc domains and FcγRs of effector cells, PF-04605412 was engineered with a point mutation in the Fc region to enhance ADCC. PF-04605412 mediates ADCC in preclinical models. PF-04605412 was well tolerated in primates up to doses of 100 mg/kg weekly.

We present the results of a first-in-human, open-label, dose-escalation study of PF-04605412 conducted at three institutions in adults with advanced solid tumors refractory to standard anticancer treatments. The starting dose of PF-04605412 for the first cohort of patients was 7.5 mg, which in a 75 kg human adult is equivalent to 1/10 of the non-observable adverse event level (NOAEL) dose in repeat-dose toxicity studies in primates (1 mg/kg qwk).

## Methods

### Study drug

PF-04605412 was supplied by the trial sponsor as a sterile solution for intravenous (IV) administration in single use 10 mL clear glass vials at a concentration of 10 mg/mL. The drug was infused in 2 h following paracetamol and antihistamine premedication. The second infusion was administered 4 weeks later and every 2 weeks thereafter.

### Trial design

Main inclusion criteria for the study included: advanced solid cancer that has progressed despite currently available therapies, Eastern Collaborative Oncology Group (ECOG) Performance Status of 0–1, adequate organ function, absence of brain metastases and no prior history of significant cardiovascular co-morbidities, bleeding disorders or vasculitis. Patients on anticoagulant therapy or taking antiplatelet agents were not eligible. Left ventricular ejection fraction and lung diffusion capacity were tested as part of the screening procedures.

The primary objective of the study was to determine the maximum tolerated dose (MTD) of PF-04605412 and recommend a dose for further clinical studies in patients with advanced solid tumors. Secondary objectives included characterization of the safety and tolerability of the drug, the PK profile and the immunogenicity of the compound and to describe any preliminary evidence of anti-tumor activity.

Dose escalation used a modified 3 + 3 design. The magnitude of increment in dose in succeeding cohorts depended on the presence of grade ≥2 drug-related toxicities. The targeted MTD was predefined as that dose level just below the dose level resulting in dose-limiting toxicities (DLTs) in ≥33 % of patients.

This study was conducted in compliance with the Declaration of Helsinki and in compliance with all International Conference on Harmonization and Good Clinical Practice Guidelines. The trial protocol and any amendments were reviewed and approved by the Institutional Review Board(s) and Independent Ethics Committee at each of the centers participating in the study.

### Safety assessments

Safety evaluations included monitoring of adverse events (AE) throughout study participation, vital signs, ECG, laboratory assessments and physical examinations at every visit. AEs were graded following National Cancer Institute Common Terminology Criteria for Adverse Events (CTCAE), Version 3.0. DLTs were defined as any grade 3 or higher AE in relation with the study drug (without a clear alternative explanation) occurring during the first 6 weeks (2 infusions) of treatment with PF-04605412.

### Pharmacokinetic analysis

Blood samples for PK analysis were drawn at prespecified time points over 24 h after the first infusion and on days 3, 5, 8, 11, 15 and 22 after initial treatment. Further samples were collected before and 2 h after each subsequent infusion. Samples were assayed by QPS, LLC (Newark, Delaware, United States) using chemiluminescence enzyme-linked immunosorbent assay methodology. Pharmacokinetic parameters by dose cohort were summarized using descriptive statistics and presented in tabular and graphical form.

### Immunogenicity and biomarker analysis

Serum samples were analyzed for anti-drug antibodies (ADA) or human antihuman antibodies using a validated analytical assay. Samples for ADA evaluation were collected just prior to each dose and monthly up to 3 months after the last administered dose. The assay used electrochemiluminescence methodology validated to detect anti-PF-04605412 antibodies in human serum samples. ADA concentration data were listed by dose level, subject and cycle. A cut-off value for antibody titers of 4.32 was considered positive.

Additional blood samples were collected at prespecified time points for exploratory assessment of changes in cells that mediate ADCC response to PF-04605412, by enumeration of natural killer (NK) lymphocytes, defined as CD16+/CD56+, and assessment of any induced cytokine response (including tumor necrosis factor alpha (TNFα), interleukin (IL)-1 beta (IL-1 β), interferon gamma (IFNγ), IL-2, IL-4, IL-5, IL-6, IL-8, IL-10 and IL-12p70).

### Anti-tumor activity

Assessment of preliminary evidence for anti-tumor activity by the Response Evaluation Criteria in Solid Tumors version 1.1 was based on CT or MRI scans performed every 6 weeks.

## Results

### Population characteristics

Thirty-three patients were treated at six dose levels. Baseline characteristics of the population are summarized in Table 1. Disease progression was the principal reason for trial drug discontinuation (*n* = 21, 63 %). The median number of cycles received was 2 (6 weeks of treatment).

**Table 1:** Patient characteristics

	*n*	%
*Gender*		
Male	18	54.6
Female	15	45.5
*Age*		
Mean (range)	56.4 years (32–74 years)	
<65 years	25	75.8
≥65 years	8	24.2
*Body mass index*		
Mean (range)	27.2 kg/m^2^ (19.8–42.0 kg/m^2^)	
*ECOG PS baseline*		
0	12	36.4
1	21	63.6
*Tumor type*		
Colorectal	9	27.2
Sarcoma	3	9.1
Head & Neck	3	9.1
Non-small cell lung	2	6.1
Cervix	2	6.1
Adenoid cystic carcinoma	2	6.1
Melanoma	2	6.1
Other	10	30.3
*Prior therapies*		
Surgery	32	97.0
Radiotherapy	22	66.7
Systemic therapy	33	100
1–2 lines of therapy	6	18.2
3 lines therapy	12	36.4
>3 lines therapy	15	45.5
*Number of metastatic sites*		
1	0	
2	7	21.2
3	14	42.4
≥4	12	36.4
Liver M1	16	48.5
Lung M1	17	51.5
Bone M1	2	6.1

### Safety and tolerability

Six dose levels were explored (7.5, 11.25, 16.9, 34, 68 and 136 mg). Twenty-three patients were evaluated for the primary endpoint (DLT and MTD). Two dose-limiting events (as defined per protocol, grade 3–4 drug-related events as per CTCAE v3.0 during the first 6 weeks of treatment) were reported: both were cases of grade 3 infusion-related reaction (hypersensitivity). One occurred in a patient receiving the lowest dose level (7.5 mg) and the other was in a patient enrolled to the highest dose cohort (136 mg). Although MTD was not achieved based on the formal DLT criteria, dose escalation was interrupted after 7 (21 %) patients in the study required permanent drug discontinuation due to a drug-related event, mainly hypersensitivity or infusion-related reactions (*n* = 5). These other infusion-related reactions were grade 2 in severity but with recurrences upon resuming drug administration. They were not considered as DLT but led to permanent discontinuation and were considered at the time of dose escalation.

Infusion-related reactions occurred across all dose levels although they were more frequent at the highest dose level tested (4/9 patients treated with 136 mg of PF-04605412). Symptoms included flushing, hypotension and nausea appearing during the first 5–10 min of drug administration. Occasionally, bronchospasm and transient grade 1–2 neutropenia and thrombocytopenia were observed. The reactions were managed with hydrocortisone and antihistamine therapy. Bronchodilators were administered when necessary. All the episodes resolved within minutes, except in the case of a 68-year-old man with metastatic non-small cell lung cancer who, after having the drug infusion interrupted and receiving appropriate treatment for the acute reaction, had a further episode of grade 3 bronchospasm and hypotension 20 min later and required a second dose of intravenous hydrocortisone. All patients were observed for an additional 8–24 h after resolution of any hypersensitivity reaction, and no residual symptoms were reported at follow-up visits.

Other than the infusion-related reactions, few grade 3 or higher adverse events secondary to PF-04605412 were observed: thrombocytopenia (*n* = 1), chills (*n* = 1) and increase of gamma-glutamyl transferase (*n* = 1). One study participant died within 30 days of last study drug administration due to progression of the underlying cancer; no deaths were reported as related to the study drug.

Table 2 summarizes the most frequently reported AEs (any grade) for all cycles. The most common AEs were fatigue (13 subjects), nausea (10), chills (8) and pyrexia (7).

**Table 2:** Drug-related adverse events of any grade observed in >1 patient

	*n*	%	7.5 mg	11.25 mg	16.9 mg	34 mg	68 mg	136 mg	Any dose level, grade ≥ 3
Patients treated	33		8	5	3	5	3	9	
Dose-limiting events			1	0	0	0	0	1	
Fatigue	9	27	1	1	2	1	1	3	0
Chills	8	24	5	1	1	0	0	1	1
Hypersensitivity/Infusion-related reaction	8	24	1	2	0	1	0	4	2
Nausea	8	24	2	1	1	1	1	2	0
Pyrexia	6	18	4	1	1	0	0	0	0
Vomiting	4	12	1	0	2	0	0	1	0
Flushing	4	12	1	1	0	0	0	2	0
Tachycardia	3	9	0	1	1	0	0	1	0
Dyspnea	2	6	0	0	0	0	0	2	1
Hypertension	2	6	1	0	0	0	0	1	0
Hypotension	2	6	1	0	1	0	0	0	0

Changes in blood cell count, presumably due to transient compartment shifts, were expected as a result of the mechanism of action of the compound. One episode of asymptomatic, transitory grade 4 thrombocytopenia was observed in a 67-year-old Caucasian man after his first cycle of treatment, with spontaneous return to baseline levels without medical intervention. The episode did not coincide with any other symptom such as an allergic reaction to study drug infusion.

Abnormalities of grade 3 or higher in the biochemistry blood tests occurred in a total of 11 patients: elevation of alanine aminotransferase, alkaline phosphatase, amylase, aspartate aminotransferase, hyperglycemia and hyponatremia. The majority of these findings were considered as not related to the study drug administration. One subject receiving 11.25 mg of PF-04605412 had asymptomatic grade 4 lipase elevation, thought secondary to the underlying cancer. Most of the laboratory abnormalities reported were grade ≤2 in severity.

### Pharmacokinetics data

PK analysis of patients across the six dose levels demonstrated a greater than dose-proportional increase of PF-04605412 Cmax, AUC_last_ and AUC_inf_ with rising doses. Inter-patient variability was high across the different cohorts, with a coefficient of variation ranging from 28–86 % to 11–90 % for AUC_inf_ and Cmax, respectively.

The volume of distribution was mainly restricted to the plasma volume. The median half-life of the drug was 36 h at the highest dose level, with a multiphasic decline in serum concentrations. Clearance was rapid and decreased with increasing dose levels (3.79 L/h at lowest dose level and 0.06 L/h at highest dose administered).

**Fig. 1:**
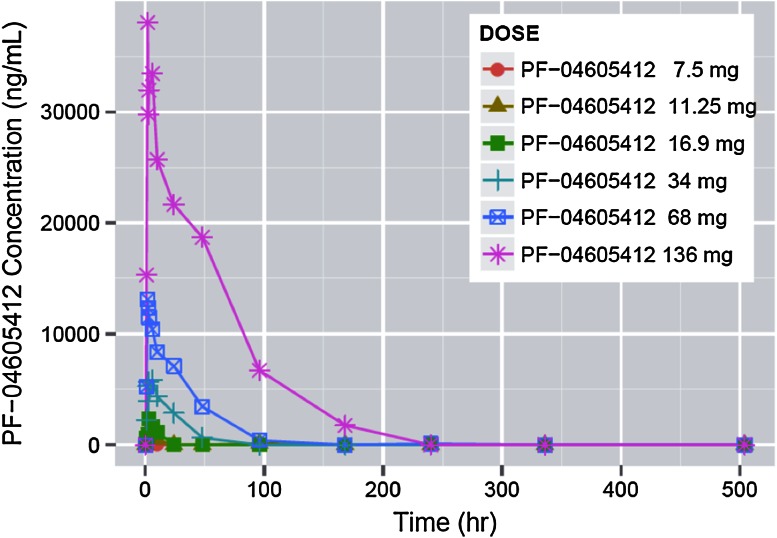
Median concentrations of PF-04605412 over time for cycle 1 by dose level

Figure 1 represents the median serum concentration of PF-04605412 over time after the first infusion, and Table 3 summarizes the main PK parameters for the dose levels tested.

**Table 3:** Geometric mean (%CV) pharmacokinetic parameters following intravenous doses of PF-04605412 (Cycle 1)

Parameters	Parameter summary statistics^a^ for PF-04605412 dose cohort					
	7.5 mg	11.25 mg	16.9 mg	34 mg	68 mg	136 mg
*N*, *n*	7, 2^b^	4	3, 2^b^	5, 4	3, 3	6, 5
*C*_max_ (ng/mL)	578.3 (37)	1,229 (27)	1,826 (90)	6,682 (11)	13,400 (18)	34,110 (37)
AUC_last_ (ng h/mL)	4,784 (171)	14,440 (170)	12,930 (103)	149,400 (71)	490,600 (85)	2,047,000 (28)
AUC_inf_ (ng h/mL)	2,000	NR	27,650	160,600 (69)	505,200 (86)	2,053,000 (28)
*t*_½_ (h)	2.70	NR	7.27	13.5 (3.99)	19.2 (8.60)	36.9 (3.96)
*T*_max_ (h)	2.00 (1.00–44.7)	3.04 (3.00–5.50)	2.43 (2.05–3.02)	2.52 (2.00–47.4)	2.00 (2.00–2.53)	6.03 (2.25–50.6)
CL (L/h)	3.79	NR	0.95	0.212 (61)	0.135 (58)	0.066 (21)
*V*_ss_ (L)	13.65	NR	10.87	6.61 (66)	5.91 (22)	3.73 (22)

The PK parameters (*t*_½_ and AUC_inf_) could not be estimated for 12 subjects due to inadequate PK profile

*N* = number of subjects; *n* = number of subjects contributing to the geometric mean for AUC_inf_, CL, and *V*_ss_

*AUC*_*inf*_ area under the plasma concentration–time profile from time 0 extrapolated to infinite time, *AUC*_*last*_ area under the plasma concentration–time profile from time 0 to the time of the last quantifiable concentration, *C*_*max*_ maximum plasma concentration, *CL* clearance, *NR* not reportable, *SD* standard deviation, *t*_*½*_ terminal half-life, *T*_*max*_ time for *C*_max_, *V*_ss_ volume of distribution, *%CV* percent coefficient of variation

^a^Geometric mean (%CV) for AUC_inf_, AUC_last_, *C*_max_, CL and *V*_ss_; arithmetic mean (±SD) for *t*_½_; median (range) for *T*_max_

^b^Geometric mean (%CV) was not reported for *n* ≤ 2, instead median was reported

According to the preclinical studies, the minimal targeted plasma concentration of PF-04605412 for therapeutic effect was 8,900 ng/mL. At the 136 mg dose level, the mean concentration of PF-04605412 fell below the projected efficacious concentration within 96 h (<15 % of the dosing interval). Based on extrapolation of drug clearance to higher doses and simulations from the available PK data, we estimated that the dose necessary to achieve this target concentration would have been at least 688 mg of PF-04605412.

### Pharmacodynamic data

#### Immunogenicity studies

Anti-PF04605412 antibodies were found in three patients (9 %) at titers of 8.15, 9.03 and 14.28 (antibody titers of 4.32 were used as cut-off value). Neither the presence nor the titer of anti-PF04605412 antibodies correlated with occurrence of infusion-related reactions and they did not appear to alter the PK profile.

#### Biomarkers of ADCC response

Sequential plasma samples from 20 patients (60 %) were suitable for assessment of changes in lymphocyte subpopulations. Results showed poor stimulation of NK (CD16+/CD56+) subpopulations at the dose levels tested, without a clear incremental effect at higher doses (Table 4).

**Table 4:** Summary of changes in CD16 +/CD56 + lymphocyte subpopulations per dose level

	Dose of PF-04605412 (*n* = evaluable patients)			
	16.9 mg (*n* = 2)	34 mg (*n* = 3)	68 mg (*n* = 1)	136 mg (*n* = 6)
*CD16* *+/CD56* *+* *cells relative to total lymphocyte count* *Mean (CV* *%)*				
Baseline	12.65 % (60.61)	20.10 % (47.88)	5.00 %	19.91 % (50.12)
Maximum change post-infusion	11.53 % (65.27)	14.72 % (43.75)	2 %	14.51 % (60.34)
Time to maximum change (h) median	2	2	2	6
*Total CD16* *+/CD56* *+* *cells/μL* *Mean (CV* *%)*				
Baseline	159.01 (100.92)	181.95 (14.64)	64	164.40 (93.88)
Maximum change post-infusion	183.25 (85.47)	199.65 (20.58)	44.0	138.39 (103.20)
Time to maximum change (h) median	85	2	1	1.5

No consistent dose-related effects on NK levels were observed. (Post-infusion value represents the value with the greatest difference from baseline. Time post-infusion indicates when this difference was observed)

#### Induced cytokine changes

The levels of TNFα, IFNγ, IL-6, IL-8, and IL-10 increased after 1–2 h post PF-04605412 infusion across all dose levels. The median time to reach peak cytokine levels (TCYTO_max_) ranged from 0.5 to 3 h across doses. Cytokine levels declined to baseline rapidly thereafter. No detectable changes from baseline were observed for IL-1 β, IL-2, IL-4, IL-5 and IL-12p70 following the administration of PF-04605412.

### Anti-tumor activity

A total of 33 patients were evaluated for the secondary endpoint of preliminary anti-tumor activity. No objective responses were observed in this study. Fourteen patients had documented progressive disease at the time of the first planned radiological assessment (week 6 of treatment). Best response of stable disease for over 24 weeks was documented for two patients, including a patient with primary adenoid cystic carcinoma who discontinued treatment after 30 weeks with stable disease due to requiring a surgical procedure for symptoms related to a benign uterine tumor.

## Discussion

This first-in-human study of PF-04605412, a fully human anti-α5β1 IgG1 mAb administered to patients with advanced solid malignancies, failed to achieve the primary endpoint. Although a formal MTD was not reached, medically significant safety events, consisting of grade 2–3 infusion reactions, were observed at multiple dose levels with a trend to worsening as the dose levels increased. Continuous evaluation of the safety events related to the drug and emerging data from real-time PK modeling, suggesting the dose necessary for eventual target modulation would be much higher than the dose levels tested, supported a joint decision by the investigators and sponsor to discontinue the clinical development of the compound.

Acute infusion-related reactions and constitutional symptoms (fatigue, nausea, chills and pyrexia) were the most common AEs and were likely related to the mechanism of action of PF-04605412. One of the five evaluable subjects (20 %) receiving 136 mg developed an adverse reaction to the infusion fulfilling the criteria to be considered a DLT, with 4 additional patients at different dose levels experiencing varying grades of acute reactions to the drug resulting in permanent discontinuation of PF-04605412.

The occurrence of constitutional symptoms is in accordance with previous reports of similar therapeutic agents [11]. Reactions to monoclonal antibodies, typically occurring acutely during the first infusion, are a well-described effect of this entire drug class, with symptoms ranging from mild rigors to systemic anaphylaxis. The rates of hypersensitivity in this study do not differ significantly from those of other mAb successfully brought into clinical practice as anticancer treatments such as cetuximab and rituximab [23]. Administration of various premedication regimens and/or adjustments to infusion duration has been used in the successful clinical development of other therapeutic mAb. In our study, however, hypersensitivity reactions proved to be the major barrier to the delivery of projected therapeutic dose levels of PF-04605412. Our attempts at intensifying the premedication regimen and modifying the duration of the infusion to improve tolerability had limited success. We were also unable to define any obvious predictive factors for the development of acute infusion reactions to PF-04605412; the presence of antidrug antibodies in 3 subjects in this study did not correlate with the appearance of the adverse reactions, similar to reports on other therapeutic antibodies [24, 25].

PF-04605412 was designed to enhance binding to Fc receptors for more effective mobilization of effector cells to mediate ADCC activity. A transient increase in cytokine production (TNFα, IFNγ, IL-6, IL-8, and IL-10) was triggered by the study drug, and temporary reductions in peripheral blood cell counts, which were an expected pharmacodynamics effect of the study drug, were observed. Changes in peripheral blood counts were possibly due to cytokine-induced margination. Interestingly, there was one case of acute and severe rapid thrombocytopenia with rapid spontaneous resolution. Bone marrow examination of this patient showed normal megakaryocytes. However, stimulation of ADCC response, indirectly evaluated based on changes in relevant peripheral lymphocyte subpopulations, was not observed at the dose levels tested in this study; a potential explanation is insufficient drug exposure to create the desired effect.

To understand better the possible causes of PF-04605412-mediated cytokine release, human endothelial cells were co-cultured with purified human PBMC in vitro in the presence of PF-04605412 (unpublished data; methods and results in supplemental material). Levels of IL-6, IL-8, IFNγ and TNFα were significantly increased in these co-cultures at 4 h after PF-04605412 treatment but these cytokines were not induced when exposing either endothelial cells or PBMC alone to the drug. Fab of PF-04605412 also did not affect the levels of cytokines in this co-culture condition. These results support the hypothesis that the cytokine levels observed in patients were the consequence of PF-04605412-mediated bridging of PBMC and endothelial cells. The short-term cytokine release observed in the clinical trial may therefore be evidence of an on-target effect of PF-04605412, which nevertheless was not associated with clinical benefit.

Pharmacokinetic assessments demonstrated more rapid drug elimination (terminal half-life 2.70–36.9 h) than expected for an IgG1 antibody. A plausible explanation for this observation is target-mediated drug disposition, which has been observed in other studies with monoclonal antibodies [26, 27]. The dose-dependent increase observed in drug clearance would support this hypothesis. Extrapolation of drug clearance at higher doses and simulations generated based on emerging pharmacokinetics data from the patients treated predict that a dose of at least 688 mg would be required to saturate α5β1 integrin on peripheral monocytes and, in turn, reach α5β1 integrin in the tumor cells in order to produce an anti-tumor effect.

Real-time evaluation of safety, pharmacokinetic and pharmacodynamics data emerging from first-in-man studies is necessary for optimal, efficient drug development. We did not find a way to prevent infusion reactions and the data emerging from PK modeling predicted that a dose 5 times above the tested dose would have been necessary to modulate target. This analysis together with a very high likelihood of re-occurring and potentially dangerous cytokine-mediated infusion reactions with escalating higher doses, made us decide to terminate the study before reaching a MTD. Moreover, no reliable signs of anti-tumor activity were observed at any of the dose levels tested. The risk/benefit assessment was therefore deemed unfavorable by the investigators and sponsor.

Early no-go decisions, as in this case, using all of the available information along with realistic and practical projections of what is likely to occur if the study continues, avoid unnecessary exposure of patients to ineffective drugs and permit optimization of all resources by academic investigators and pharmaceutical industry.

## Conclusions

In this trial, we were not able to achieve a formal MTD. Dose escalation was halted by the investigators and sponsors taking the following factors into account: grade 3 toxicity (acute infusion-related reactions) that could not be mitigated by standard measures in the outpatient clinic (including changes in infusion duration and various types of premedication regimens), the observation that 4 of 9 patients at the highest dose level tested developed clinically significant reactions, and PK modeling that indicated that we would need to escalate the dose fivefold more in order to achieve predicted target dose levels for target saturation.

This trial highlights the value of vigilant toxicity monitoring and real-time PK data and modeling. In the face of medically significant events thought directly related to the mechanism of action of the drug, and therefore likely to be dose-related, further dose escalation was not thought to be feasible without placing patients at unacceptable risk. Therefore, further clinical development of PF-04605412 is not recommended.

## Electronic supplementary material

Below is the link to the electronic supplementary material.

Supplementary material 1 (DOCX 1049 kb)
