# Supplementary material for: A first-in-human study of the anti-α5β1 integrin monoclonal antibody PF-04605412 administered intravenously to patients with advanced solid tumors
Source: Cancer Chemother Pharmacol. 2014 Sep 12;74(5):1039–46. doi: 10.1007/s00280-014-2576-8 (PMC4209234; doi:10.1007/s00280-014-2576-8)

**Supplemental table 1**. Complete list of eligibility criteria for participation in the study.

***Inclusion criteria:***  Patients had to meet all of the following inclusion criteria to be eligible for enrollment into the trial:

- Patients with histologically or cytologically confirmed advanced measurable or evaluable solid tumors unresponsive to currently available therapies, or for which there is no curative therapy.
- Eastern Collaborative Oncology Group (ECOG) performance status 0-1.
- Life expectancy ≥12 weeks.
- Age ≥18 years.
- Adequate bone marrow function as defined by:
  - Absolute neutrophil count (ANC) ≥1500/μL;
  - Platelets ≥100,000/μL;
  - Hgb >9 g/Dl;
  - Albumin ≥30 g/L.
- Adequate liver function as defined by:
  - Total bilirubin ≤1.5 x UNL;
  - AST/ALT and ALK phosphatase ≤2.5 x ULN or ≤5 x ULN with documented liver  and/or bone metastases.
- Adequate renal function as defined by:
  - Serum creatinine ≤1.5 mg/dL or calculated creatinine clearance or measured >60 ml/min;
  - Dipstick urine protein <2+ or dipstick urine protein >2+ but with <500 mg urinary protein/24 hours.
- Resolution to baseline (or ≤Grade 1 for neuropathy or hand-foot syndrome) of any toxic side effects related to prior chemotherapy, radiation therapy, biologic therapy, or hormonal therapy, except alopecia.
- Female patients must be surgically sterile or be postmenopausal, or must agree to use effective contraception during the period of therapy and for 6 months after discontinuing study treatment. All female patients with reproductive potential must have a negative pregnancy test (serum or urine) within the 7 days prior to enrollment. Male patients must be surgically sterile or must agree to use effective contraception during the period of therapy and for 6 months after discontinuing study treatment. The definition of effective contraception should be in agreement with local regulation and based on the judgment of the principal investigator or a designated associate.
- Signed and dated informed consent indicating that the patient has been informed of all the pertinent aspects of the trial prior to enrollment.
- Ability and will to comply with the study scheduled visits, treatment plans, laboratory tests and other procedures.
- In addition, patient, to be enrolled in the pharmacodynamic cohort (1B) , must also meet the following criteria:
  - No contraindication to DCE-MRI scanning;
  - Ability to hold breath for at least 30 seconds;
  - Access for intravenous contrast injection;
  - Lesions eligible for DCE-MRI scanning and detectable enhancement;
  - Expression of integrin α5β1 (either obtained on archival tissue specimen or fresh  biopsy).

***Exclusion criteria;***  Subjects presenting with any of the following were not be included in the trial:

- Known brain metastasis.
- Chemotherapy, radiotherapy, or any investigational cancer therapy within 4 weeks of start of screening procedures.
- Any surgical procedure within 4 weeks of start of screening procedures.
- Active bleeding disorder, including gastrointestinal bleeding, as evidenced by hematemesis, significant hemoptysis or melena in the past 6 months.
- Any history of myocardial infarction, severe/unstable angina, coronary/peripheral artery bypass graft, congestive heart failure, cerebrovascular accident including transient ischemic attack, or pulmonary embolus, thromboembolic event or peripheral vascular disease.
- QTc prolongation defined as >470 msec.
- Active or chronic Hepatitis B or C.
- Severe COPD, asthma or conditions that impair blood gas diffusion (eg, Lung Diffusion Capacity Testing at Screening ≤50%).
- Autoimmune disorders (eg, Crohn’s Disease, rheumatoid arthritis, scleroderma, systemic lupus erythematosus) and other diseases that compromise or impair the immune system.
- Left Ventricular Ejection Fraction (LVEF) <40%.
- History of acute renal failure related to glomerular or vascular injury.
- Uncontrolled diabetes.
- Active peptic ulcer disease requiring treatment in the past 6 months.
- Uncontrolled hypertension defined as baseline systolic blood pressure readings more than 145 mmHg, and baseline diastolic blood pressure readings more than 90 mmHg (patients whose hypertension is controlled by antihypertensive therapies are eligible).
- Known HIV infection.
- Previous history of vasculitis.
- Known or suspected right to left or bidirectional, or transient right to left cardiac shunts. Screening echocardiography will be performed on patients with suspected shunts (applicable only to patients undergoing optional evaluation by ultrasonography with microbubbles).
- Requirement of anticoagulant therapy except for low-dose anticoagulants for maintenance of patency of central venous access or prevention of blood clotting.
- Current use of antiplatelet agent (eg, clopridogel or ticlopidine).
- Administration of concomitant immune suppressive drugs.
- Biologic or immunotherapy <4 weeks or 5 half-lives prior to enrollment, whichever is longer.
- Pregnancy or breastfeeding.
- Known or suspected hypersensitivity to perflutren, octafluorpropane or to any of the other ingredients in DEFINITY® or other microbubble contrast agent (applicable only to patients undergoing ultrasonography with microbubbles).
- Other severe acute or chronic medical or psychiatric condition, or laboratory abnormality that may increase the risk associated with study participation or study drug administration, or may interfere with the interpretation of study results, and in the judgment of the investigator would make the patient inappropriate for entry into this study.

**Supplemental Figure 1**. **Co-culture experiments of human umbilical vein endothelial cells (HUVECs) and purified human peripheral blood mononuclear cells (PBMC) in vitro in presence of PF-04605412 (PF-5412)**. Results suggest that the PF-04605412-mediated interaction of PBMCs and human endothelial cells may have contributed to increased levels of cytokines in patients.

- *Materials* Cells: HUVEC (Lonza, MD,), human PBMC (purified from healthy donor, AA158: V/V). Antibodies: PF-5412 (anti-human integrin α5 IgG1/DLE) , Fab of PF-04605412, 22B5IgG1wt (anti-human integrin α5 IgG1), 22B5IgG4wt (anti-human integrin α5 IgG4), anti-KLH human IgG1/DLE. Human cytokines multiplex kit (EMD Millipore, MA, cat#HCYTOMAG-60K-06): IL-2, IL-6, IL-8, IL10, TNFα, INFγ.
- *Methods***:** HUVEC (4e5/well) were seeded into 6-well plates and cultured overnight. Purified human PBMCs (2e6 cells in 250µl medium) were added to each well. PF-04605412, control mAbs, and/or vehicle (PBS) were then added to a final concentration of 50 μg/ml. Supernatants were collected at 0.5h, 2h, and 4h after culture at 37^o^C, and clarified by centrifugation at 5000rpm for 10 minutes at 4^o^C. Cytokine levels were determined by multiplex assay following the manufacturer’s instructions.
- *Results* Levels of IL-6 (graph A), IL-8 (graph B), TNF-α (graph C) and INF-γ (graph D) were significantly increased in co-cultures of endothelial cells (EC) and human PBMC at 2h (left column) and 4hr (right column) after PF-5412 treatment. PF-5412 did not affect cytokine release by endothelial cells or PBMC alone. Fab of PF-5412 and anti-human integrin a5 IgG4 (with identical epitope of PF-5412) also did not affect the levels of cytokines in these co-culture conditions.


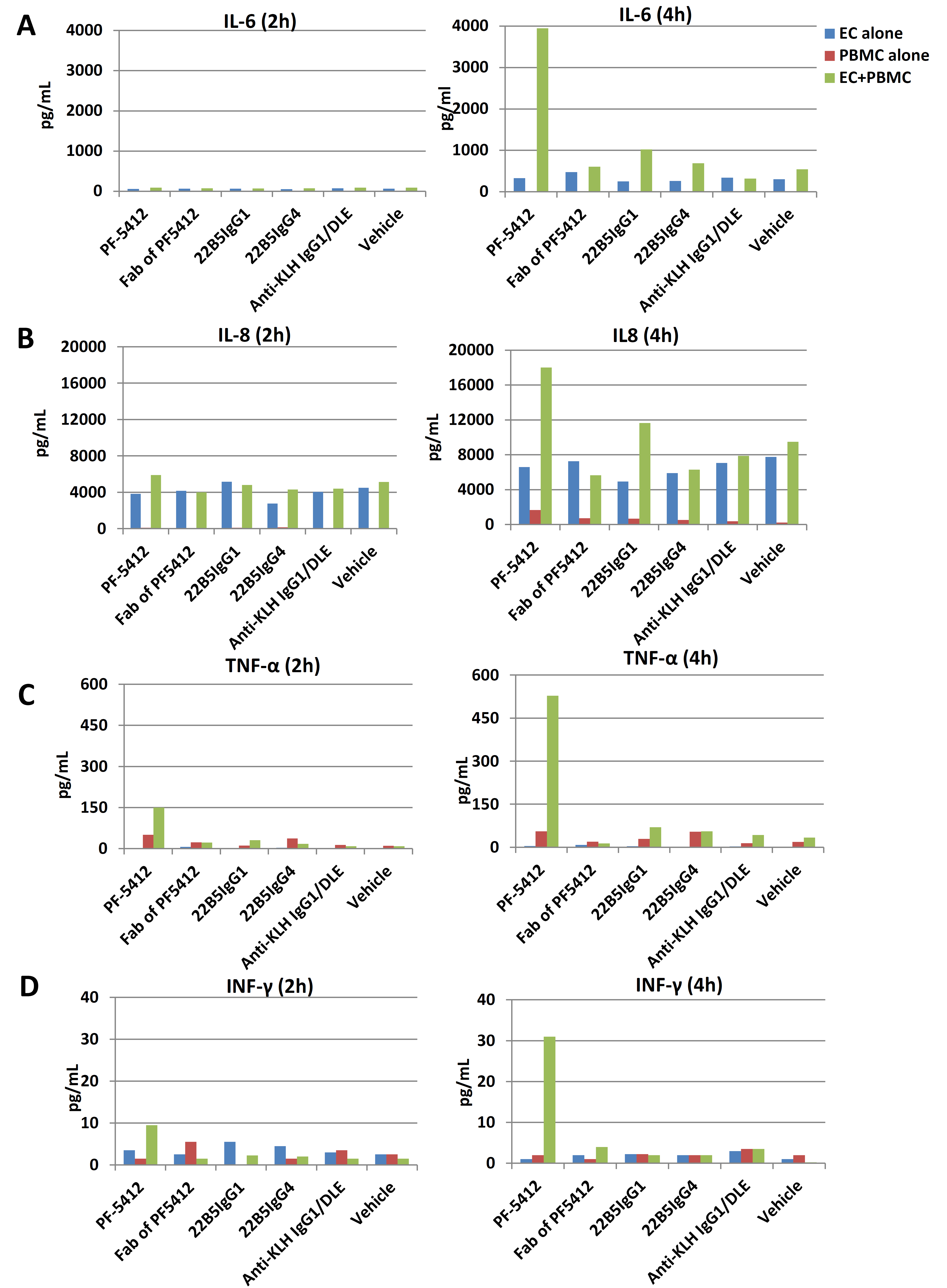

Supplement: Supplementary file 1 — Supplementary material 1 (DOCX 1049 kb) [file 280_2014_2576_MOESM1_ESM.docx]
